# Effect of Stereolithography 3D Printing on the Properties of PEGDMA Hydrogels

**DOI:** 10.3390/polym12092015

**Published:** 2020-09-03

**Authors:** Gavin Burke, Declan M. Devine, Ian Major

**Affiliations:** Materials Research Institute, Athlone Institute of Technology, Dublin Road, N37 HD68 Co. Westmeath, Ireland; g.burke@research.ait.ie (G.B.); ddevine@ait.ie (D.M.D.)

**Keywords:** PEGDMA, stereolithography, 3D printing, UV chamber curing, photopolymerisation

## Abstract

Stereolithography (SLA)-based 3D printing has proven to have several advantages over traditional fabrication techniques as it allows for the control of hydrogel synthesis at a very high resolution, making possible the creation of tissue-engineered devices with microarchitecture similar to the tissues they are replacing. Much of the previous work in hydrogels for tissue engineering applications have utilised the ultraviolet (UV) chamber bulk photopolymerisation method for preparing test specimens. Therefore, it is essential to directly compare SLA 3D printing to this more traditional approach to elucidate the differences in hydrogels prepared by each fabrication method. Polyethyleneglycol dimethacrylate (PEGDMA) is an ideally suited material for a comparative study of the impact that SLA fabrication has on performance, as the properties of traditional UV chamber-cured hydrogels have been extensively characterised. The present study was conducted to compare the material properties of PEGDMA hydrogels prepared using UV chamber photopolymerisation and SLA 3D printing. From the subsequent testing, SLA-fabricated hydrogels were shown to maintain similar thermal and chemical performance to UV chamber-cured hydrogels but had a higher compressive strength and tensile stiffness, as well as increased hydrophilicity. These differences are attributed to the increased exposure to UV light SLA samples received compared to traditionally UV chamber-cured samples.

## 1. Introduction

Ultraviolet (UV) curing is a chemical reaction by which a polymerisable monomer or macromolecular monomer is exposed to UV radiation in the presence of a photoinitiator to form crosslinks. The early scientific work for the UV polymerisation process was conducted during the 1940s by several chemical companies including Dupont and Monsanto, and further improved upon over the next two decades [1,2,3,4]. UV chamber curing is an advantageous technique as it allows for control over the spatial and temporal parameters of curing and thus has been a greatly utilised method in the fabrication of hydrogels. However, it also suffers from several disadvantages, such as oxygen inhibition and the potential for the unreacted monomer to be present post-cure [5,6]. Final shape geometries were also limited to those that could be prepared from a mould and the necessity for light to be evenly spread across the monomer surface, indicating a lack of complexity in most UV chamber-fabricated samples, limiting their potential for tissue engineering applications.

An emerging technology which has shown the potential to advance polymer-based tissue-engineered devices is the 3D printing technique stereolithography (SLA) [7,8,9]. This technique has proven to have several advantages over traditional polymerisation techniques. SLA permits for the control of curing at resolutions as low as 27 µm [10], allowing for the possibility to create tissue-engineered devices with microarchitecture similar to the tissues they are replacing. SLA also gives greater control over the porosity of the hydrogel, a factor which can play a significant role in determining the differentiation and function of cells [11,12]. The SLA process solidifies a liquid resin through exposure to a UV light, allowing SLA photopolymerisation to take place at room temperatures, which provides SLA with advantages over temperature-reliant processes including injection moulding and extrusion, as temperature-labile drugs risk significant degradation by these processes [13,14]. Unlike conventional UV chamber curing, the SLA process occurs when a laser causes a localised solidification of photocrosslinkable polymers to create a solid layer. Following the solidification of the first layer, the platform moves to allow a new layer to be cured on top [8,15]. A common limitation of this layer-by-layer approach to photocuring is the potential to leave areas of the forming design under or over-polymerised, thus affecting the properties of samples prepared by this method. In order to counteract this effect, a post-cure of the polymer is typically carried out [16]. However, this runs the risk of over-curing already polymerised sections which can be detrimental to the mechanical properties of the polymer [15].

The mechanical properties of a polymer are an important consideration for determining their potential uses in tissue engineering applications [17,18], with different tissues having mechanical strengths varying from 11.7 MPa for nerve tissues to 35.7 MPa for arterial cartilage to 170–193 MPa for cortical bone [19,20,21]. Thus, there is a necessity to determine the range of mechanical strength into which a polymer is categorised. Similarly, the stiffness of a matrix upon which cells are attached in tissue engineering is a very important factor for both cell proliferation and cell differentiation, considered of similar importance to the incorporation of biological signalling [22,23,24]. Other material properties such as wettability are an important consideration for biomedical applications as they can have a significant impact on the ability of cells to attach to a polymers’ surface with ideal cell adhesion occurring with contact angle measurements between 40° and 60° [25]. With the need for polymers to maintain stability at biologically relevant temperatures, it is important to understand the potential impacts SLA may have on the thermal transitions of a polymer. If SLA is to continue being utilised in tissue engineering applications, it is necessary to understand the potential impact of SLA on these material properties of polymerised hydrogels compared to UV chamber-polymerised hydrogels.

There have been numerous hydrogels which have received interest due to their properties for tissue engineering applications including chitosan, hyaluronic acid (HA) and poly-l-lactic acid (PLA) [26,27,28]. Hydrogels come with their own advantages, including being easily modifiable and good transporters, and disadvantages such as a typically low mechanical strength and difficulty in handling. Polyethylene glycol (PEG) based hydrogels including polyethyleneglycol dimethacrylate (PEGDMA) have been used extensively in various tissue engineering applications as they have well characterised material properties, lack of immunogenicity, recognised cytocompatibility, and ease of synthesis [29,30]. Additionally, PEGDMA material properties can be easily controlled through modifications in chain length, alterations to the weight percentage of the macromer per hydrogel, and via the addition of other materials to form a hydrogel composite. Although PEGDMA and similar hydrogels have numerous advantages and have seen a great deal of interest to date, its use as a base for the 3D printing technique SLA has been focused more on the viability of the 3D printing technique [15,31,32,33], whereas the impact SLA has on the material properties of PEGDMAs is yet to be fully explored. The present study was conducted to compare the material properties of a PEGDMA hydrogel prepared using the conventional UV chamber curing compared to the less conventional 3D printing technique SLA with the purpose of elucidating differences in the performance in hydrogels by the two fabrication methods so to permit other researchers in making changes in material formulation accordingly. This study will act as a basis for the future use of PEGDMA in SLA-based applications and provide a model polymer with the potential to assist in predicting the impact of SLA on similar polymers.

## 2. Materials and Methods

### 2.1. Materials

The macromolecular monomer, polyethyleneglycol dimethacrylate M_W_ 600 was purchased from Polysciences (Polysciences Gmbh, Hirschberg an der Bergstrasse, Germany). The photoinitiators used were Irgacure 2959 supplied by Ciba Specialty Chemicals (Basel, Switzerland) for the UV chamber, and diphenyl(2,4,6-trimethylbenzoyl)phosphine oxide (TPO) purchased from Tokyo Chemical Industry UK Ltd. (Oxford, UK) for SLA. The 3D printing machine utilised was the Formlabs Form 2 SLA (Formlabs Inc., Somerville, MA, USA). All materials were used as received.

### 2.2. Hydrogel Fabrication

PEGDMA monomers were photopolymerised using a UV curing system (Dr. Gröbel UV-Electronik GmbH) in an irradiation chamber with a controlled radiation source consisting of 20 UV-tubes in the spectral range of 315–400 nm at an average intensity of 10–13.5 mW/cm^2^. The mixtures were prepared pre-polymerisation by combining PEGDMA with either 1 wt% Irgacure 2959 photoinitiator or TPO. The UV chamber batches were prepared as previously established [34], being placed in a 50 mL beaker, mixed using a magnetic stirrer for 2 h until a homogenous mixture was achieved. The solutions were then pipetted into silicone moulds, and photopolymerisation was carried out for 10 min, after which time gelation had occurred. The SLA batches were prepared in a 500 mL beaker and stirred as per above. Following the mixing, the SLA monomer mix was added to a form 2 resin tank and printed on a Form 2 SLA 3D printer utilising a 250 mW laser at a 405 nm spectral wavelength. Samples were printed with a layer thickness of 50 um and then post-cured using a Procure 350 UV chamber (3D systems) for 10 min. For both SLA and UV chamber-polymerised PEGDMA, tensile samples were prepared to match the dimensions specified for a ASTM Type IV test specimen (D 638-02a). Large discs for compressive testing and wettability analysis with sample dimensions of 23 mm diameter × 2.2 mm thickness and small discs with 1.6 mm diameter × 1.0 mm thickness for Swelling studies, differential scanning calorimetry and Fourier transform infra-red spectroscopy, were prepared. Following photopolymerisation, the samples were stored in 50 mL universals containing phosphate buffer solution at 37 °C until testing.

### 2.3. Material Property Characterisation

#### 2.3.1. Swelling Studies

Swelling studies were carried out on PEGDMA samples prepared via UV chamber and SLA polymerisations. Samples tested in quintuplicate (n = 5) were weighed and placed in McCartney bottles filled with phosphate buffer solution (PBS) (pH 7.4). Once equilibrium swelling was reached, the samples were weighed again and the swelling and gel fraction values were calculated as per Equations (1) and (2) below—where *W_s_* and *W_d_* are the weights of the hydrogels in the swollen state and the dried state, respectively, and *W_rd_* is the hydrogel weight following re-dry:

(1) $$Swelling\;ratio\;\left(\%\right)=\left(\frac{W_{s}-W_{d}}{W_{d}}\right)\times \frac{100}{1}$$

(2) $$Gel\;fraction=\left(\frac{Wrd}{Wd}\right)\times \frac{100}{1}$$

#### 2.3.2. Chemical Analysis

The confirmation of polymerisation was determined using attenuated total reflectance Fourier transform infrared spectroscopy (ATR-FTIR, Perkin Elmer), and samples were stored in a vacuum oven at 80 °C prior to testing. Samples were analysed at room temperature in the spectral range of 4000–650 cm^−1^ with 4 scans per sample and a constant compression force of 80 N (n = 3). The confirmation of polymerisation was based on the disappearance of peaks at 815 cm^−1^ and 1167 cm^−1^ associated with C=CH and C–O bonding as was previously established [34,35].

#### 2.3.3. Dynamic Mechanical Analysis

To determine the tensile strength of samples and characterise the differences in thermal behaviour, tensile and thermal DMA testing was carried out using a TA Q-800 fitted with a Peltier temperature control. For the tensile testing, the temperature was controlled at 37 °C and the samples were tested at a displacement rate of 0.1 mm/min. Tensile strength at limit and Young’s elastic modulus were calculated from the subsequent stress/strain curves. For the thermal analysis, the samples were cooled to approximately −70 °C and exposed to a static oscillatory force of 0.01 N at a constant frequency of 1 Hz, while being heated at a rate of 3 °C/min. From these conditions, both the storage/elasticity (G′) and loss (G″) moduli were obtained for both PEGDMA preparations.

#### 2.3.4. Compression Testing

The compressive strength at limit and compressive moduli were established using a Lloyd Lr10K screw-driven mechanical testing machine as per our previous study [35]. Cylindrical samples with average diameters and heights of 28 mm and 2.5 mm, respectively, were equilibrated at room temperature prior to testing. Unconfined compression was subsequently carried out at a speed of 1 mm/min. Samples were compressed to 60% strain with a pre-load of 5 N.

#### 2.3.5. Wettability Measurement

The wettability of samples was determined by the sessile drop method using an FTA 1000 class B Goniometer (First ten Angstroms), 10 μL of deionised water was placed on the surface of the polymer, and the contact angle of the droplet was measured. Contact angles were recorded in three different areas of each hydrogel (n = 3), with 3 hydrogels examined for both the UV chamber and the SLA-based polymerisation.

#### 2.3.6. Thermal Properties

Differential scanning calorimetry was conducted to determine whether there was a difference in thermal transitions following different polymerisation processes. Hydrogels to be tested were dried in a vacuum oven with 50 mBar of pressure at 80 °C overnight. Samples with a weight between 8 and 12 mg were weighed using a Sartorius microbalance, heated to 200 °C at a rate of 20 °C/min and cooled to −50 °C using the cooling compartment of a modulated differential scanning calorimetry (DSC) machine. Samples were then heated at a rate of 5 °C/min to 200 °C. Glass transition temperatures were calculated using the resulting thermographs (n = 2). The calibration of the instrument was carried out using indium any volatiles were purged using nitrogen gas at a rate of 30 mL/min.

#### 2.3.7. Statistical Analysis

Data are expressed as the means ± standard error of the mean. Differences between groups were analysed using unpaired T-tests with 95% confidence intervals. A *p*-value of less than 0.05 was considered statistically significant (* *p* < 0.05, ** *p* < 0.01 and *** *p* < 0.001). All data analysis was carried out using Graphpad Prism 7.

## 3. Results

### 3.1. Swelling Characteristics

**Figure 1:**
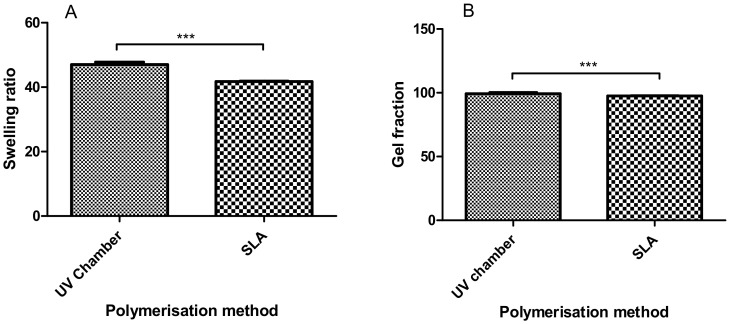
Comparison between (**A**) the swelling characteristics and (**B**) the equilibrium water content of the UV chamber-polymerised polyethyleneglycol dimethacrylate (PEGDMA) hydrogels and the stereolithography (SLA)-polymerised PEGDMA hydrogel.

Hydrogel swelling ratios provide an indication of how tightly polymer networks are cured, with greater swelling being associated with a greater freedom between polymer chains. In Figure 1A, the swelling ratios of the UV chamber and SLA samples were shown to be significantly different at 47.0 and 41.7, respectively, indicating that the SLA-fabricated hydrogels have less chain freedom compared to the UV chamber samples. With respect to the gel fraction results in 1(B), both processes resulted in an unreacted monomer percentage below 5%, with results of 99.33% and 97.58% for the UV chamber and SLA samples, respectively.

### 3.2. Chemical Analysis

**Figure 2:**
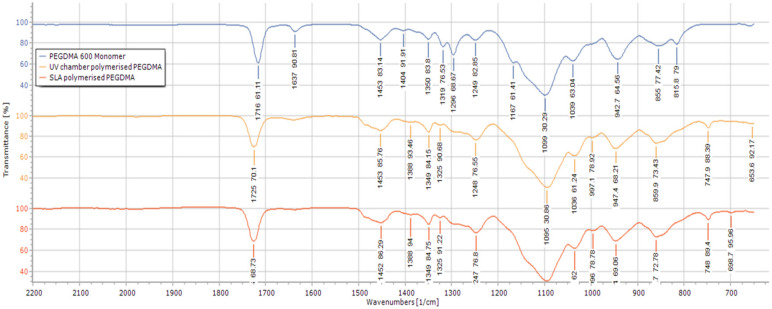
FTIR spectra of the UV chamber-polymerised PEGDMA hydrogels, SLA-polymerised PEGDMA hydrogels and the unpolymerised PEGDMA monomer.

To further confirm that the curing was completed, FTIR analysis was carried out as shown in Figure 2. Both the UV chamber- and SLA-fabricated hydrogels had peaks at 815 cm^−1^ and 1167 cm^−1^, indicative of C=CH bending and C–O bonding, respectively [36,37,38], when compared with the FTIRs of the PEGDMA monomer. There was a small peak present at 1637 cm^−1^ associated with CH=CH bonding [39], highlighting the presence of unreacted monomer in both the UV chamber and SLA-fabricated hydrogels. This agrees with what was seen in the gel fraction results wherein less than 3% of the PEGDMA was present in both the UV chamber and the SLA-fabricated hydrogels remained unreacted.

### 3.3. Dynamic Mechanical Analysis

**Figure 3:**
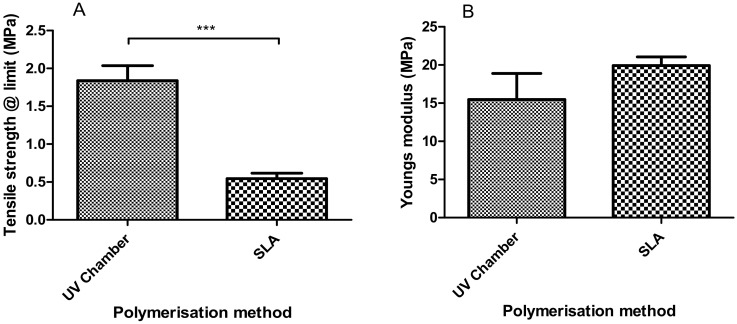
Dynamic mechanical analysis results showing (**A**) the tensile strength at limit and (**B**) Young’s modulus of the UV chamber-polymerised PEGDMA hydrogels and the SLA-polymerised PEGDMA hydrogels.

From the tensile testing results (Figure 3), the SLA samples were shown to have slightly higher tensile stiffness when compared with the UV chamber samples with Young’s modulus values of 15.47 MPa and 19.92 MPa, respectively. These values are not mirrored with tensile strength at limit where the UV samples were shown to have almost four times the tensile strength of SLA-fabricated samples with values of 1.84 MPa and 0.54 MPa, respectively.

**Figure 4:**
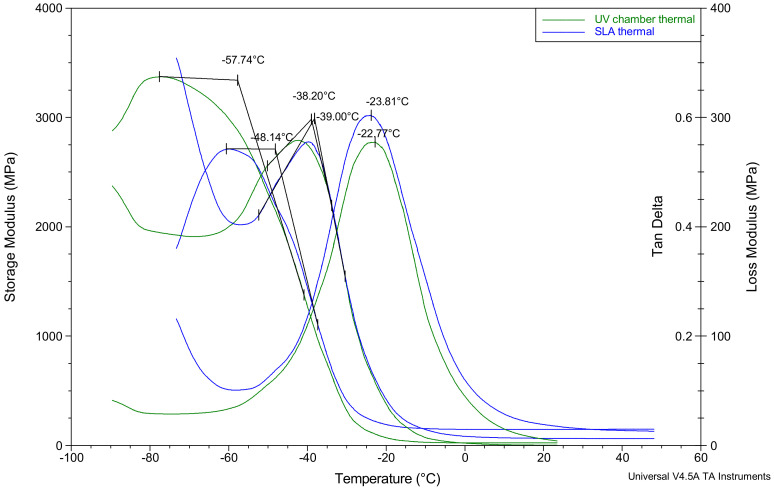
Dynamic mechanical analysis results showing the storage modulus (G’), the loss modulus (G’’) and the tan delta for both PEGDMA samples prepared via UV chamber and PEGDMA hydrogels prepared by SLA.

The thermal analysis results shown in Table 1 and Figure 4 provide a similar outlook regarding the differences in stiffness values between the UV chamber and SLA samples. Based on the storage modulus measurements, there was a decrease in the stiffness for UV chamber hydrogels at −57.7 °C whereas with SLA hydrogels, the same decrease was not seen until −48.1 °C, indicating a higher degree of stiffness in the SLA samples compared to the UV chamber samples. The peak of loss modulus values for both the UV chamber and SLA samples were much closer, being −38.2 °C and −39.0 °C, respectively, and the mean tan δ values similarly were close, being within 1.5 °C of one another. With the tan δ peak corresponding more closely to a glass transitions midpoint than its beginning, and the loss modulus peak corresponding more closely to the beginning of the glass transition [40], it appears that the thermal behaviour of the PEGDMA hydrogels were not affected by the change in curing process.

### 3.4. Compression Testing

**Figure 5:**
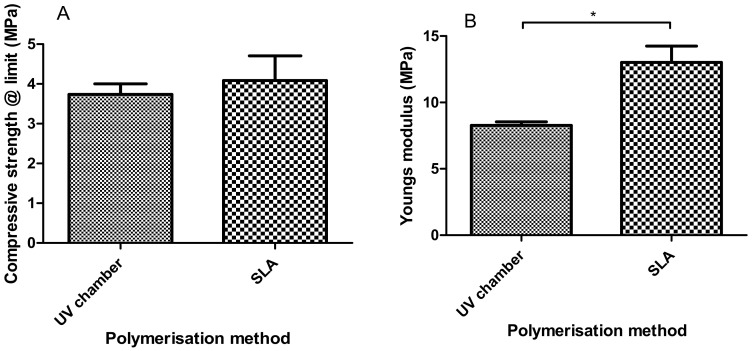
Compressive properties of PEGDMA (UV chamber) and PEGDMA (SLA) hydrogels showing (**A**) the3 compressive strength at limit and (**B**) Young’s modulus.

Similarly to what was seen with tensile Young’s modulus measurements in Section 3.3, the compressive Young’s modulus values shown in Figure 5 had SLA hydrogels with increased stiffness values when compared to the UV chamber samples; with Young’s modulus values of 13.01 MPa and 8.26 MPa, respectively. Moreover, the compressive strength values of 3.73 MPa for the UV chamber samples and 4.08 MPa for the SLA samples were also observed with no significant differences showcasing the comparability of the overall strength of the PEGDMA samples.

### 3.5. Wettability Measurements

**Figure 6:**
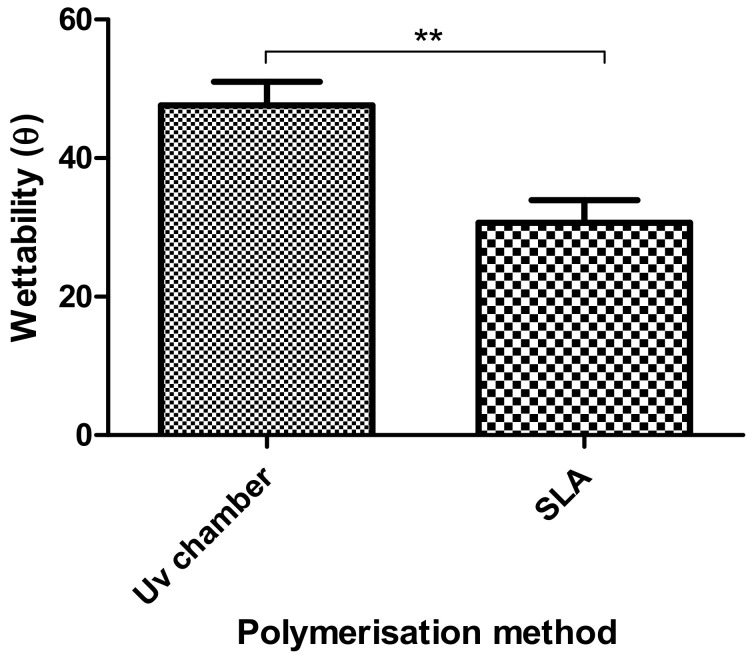
Wettability measurements of the UV chamber-based PEGDMA hydrogels and the SLA-based PEGDMA hydrogels.

In Figure 6, the wettability of the UV chamber and SLA hydrogels were compared. From Figure 6, it could be seen that while both PEGDMA hydrogels were hydrophilic (contact angle less than 90°), the SLA-polymerised PEGDMA samples (30.6°) were significantly more hydrophilic than the UV chamber-polymerised PEGDMA.

### 3.6. Thermal Properties

**Figure 7:**
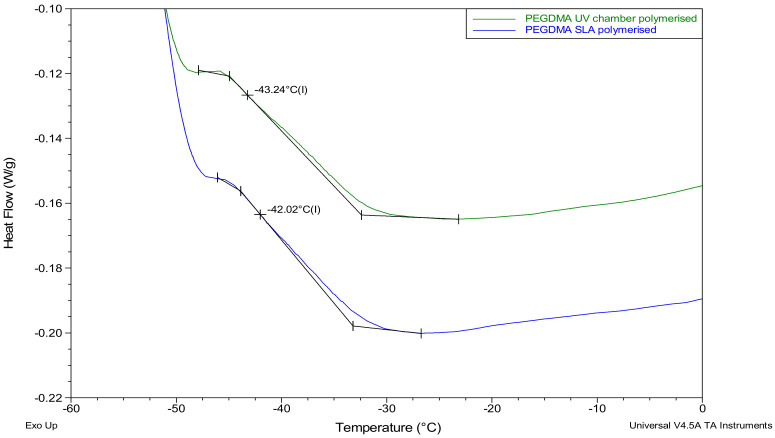
Differential scanning calorimetry (DSC) thermograms highlighting the glass transition temperatures for the PEGDMA samples prepared via UV chamber and SLA polymerization.

The thermal transitions of the UV chamber- and SLA-polymerised PEGDMA samples were found to be within a degree of one another, as shown in Table 2. Similar comparability was seen in Section 3.3 where the glass transition temperatures based on the loss modulus and tan delta were also found to be within a degree of one another. Confirming the results seen with the loss modulus and tan delta in Section 3.3, the glass transition temperatures were found to be within a degree of each other, and the UV chamber samples Tg values were slightly lower than the SLA samples, at −42.5 °C and −41.7 °C, respectively (Figure 7).

## 4. Discussion

In this study, a side-by-side comparison of the properties of UV chamber-cured and SLA 3D printed PEGDMA hydrogels was made. The swelling ratio results shown in Figure 1A showed that the SLA-polymerised PEGDMA had a significantly lower swelling ratio when compared to the UV chamber samples. This indicated that a more tightly crosslinked polymer network was found in the SLA-polymerised samples [41,42]. This was further indicated through the analysis of both the thermal characterisations and wettability measurement. In Section 3.3, the glass transition temperatures, indicative of the temperature needed to achieve the chain relaxation, were slightly higher in the SLA samples, a result of which was repeated in Section 3.6. However, this difference was minor (being less than 1.5 °C in difference), which was nonetheless surprising as the increased polymer stiffness (shown in Figure 3B and Figure 5B) of the SLA samples would be expected to necessitate increased thermal energy to undergo chain relaxation. The lack of discernible differences in the thermal properties matched more closely with the expected outcome of this study, wherein there would be relatively minor changes in properties: despite the process of polymerisation being different, their underlying mechanisms remain the same. Regarding wettability, it was thought that the layer-by-layer polymerisation process would create minor imperfections on the surface of the SLA samples causing a decrease in overall wettability compared to UV chamber samples [43]. This was confirmed with UV chamber samples having a wettability measurement of 47.6° significantly less hydrophilic than SLA samples at 30.6°. It is likely that further hydrogels prepared through SLA fabrication will experience a similar shift in wettability measurements, becoming more hydrophilic than the UV chamber-cured counterparts. This may prove beneficial for the use of more hydrophobic photopolymers such as itaconic acid-based polyesters or epoxidized soybean oil [44,45].

Considering swellability is higher in the less hydrophilic UV chamber samples than the more hydrophilic SLA samples, it would indicate a greater degree of chain freedom in the UV chamber samples. Furthermore, the analysis of mechanical testing also indicates that there was greater chain freedom in UV chamber samples as they were shown to be significantly less compressively stiff. From these results, it could be assumed that there was a greater degree of curing in the SLA 3D printed samples, which however was not backed up by either chemical analysis or gel fraction results. Both FTIR and gel fraction highlight the high degree of curing efficiency in both fabrication processes. Interestingly, the gel fraction results imply that although the SLA-polymerised samples were shown to have a high degree of polymerisation efficiency, they were also of a lower efficiency than the UV chamber samples, with approximately four times the amount of gel fraction present in SLA hydrogels compared to the UV chamber samples (0.67% compared to 2.42%). Under these circumstances, it appears that despite having less polymerised material, the SLA-polymerised samples have a greater degree of crosslinking density, impacting on the polymer chain movement. It should be noted that most SLA curing processes, including the one described above, have a post-cure process wherein the samples which have been printed are cleaned of unreacted monomer and placed into a UV chamber for 10 min, effectively giving these samples a second curing. There is potential for this approach to produce several of the above differences due to the over-curing of the polymer, however this is unlikely to be the case, as upon polymerisation the samples were not subject to the warping that would typically be associated with the over-curing of a hydrogel [15].

Another potential cause of these differences in properties can be assumed based on the mechanics of the SLA fabrication process, namely that structural imperfections exist throughout SLA-fabricated hydrogels, caused by the nature of its layer-by-layer curing process. Through this layer-by-layer curing, localised regions of unreacted monomer are formed which affect the overall strength of the hydrogel [16]. In theory, the UV chamber exposure would polymerise these pockets of unreacted monomer; however, there is credence lent to this theory by the slightly increased presence of unreacted monomer in SLA samples, as outlined in Section 3.1. By examining the differences between the compressive and tensile results, it would be thought that the higher crosslinking density indicated by swelling and thermal analysis would lead to higher tensile strengths in the SLA samples [46]. This is true of the tensile stiffness values shown in Section 3.3, however, it does not occur for tensile strength at limit where although both samples have much lower tensile strengths compared to tensile stiffness, the SLA-polymerised samples were significantly weaker than the UV chamber-polymerised samples.

A more likely cause of the contrast in the expected tensile properties may be due to the print direction of samples. All samples were printed based on Form2 software recommendations, with uniform x, y and z orientations. It has been established by others that print orientation, particularly z-orientation, has an impact on the mechanical properties of 3D printed components [47]. The work of Mueller described identical outcomes to our study, wherein the tensile stiffness was minimally impacted by the print direction due to the effect not occurring until the bonds were broken, whereas there was a drastic decrease in the tensile strength [48]. Considering the likely impact of z-orientation on tensile strength, it is likely that SLA conducted under different z-orientations would demonstrate higher tensile strengths being comparable or greater than UV chamber samples predicted by the compressive strength results seen in Figure 5A. In contrast to the tensile results, compressive Young’s modulus and compressive strength at limit values are relatively close in value to one another with SLA samples experiencing a smaller drop off from their stiffness value. Moreover, they maintain higher compressive strength values than UV chamber samples which would be expected with greater crosslinking densities. This is an important consideration when looking to advance SLA-prepared hydrogels for tissue engineering applications, as pockets of unreacted monomer could result in subpar or unpredictable mechanical performances in otherwise well characterised materials.

## 5. Conclusions

This study found that both polymerisation methods were successful in forming PEGDMA-based hydrogels with less than 3% of PEGDMA monomer from either process being unreacted. No significant impact on the thermal properties of the samples was noted, nor was there a difference in the chemical properties between the samples. The wettability of PEGDMA samples was found to be significantly increased in the SLA 3D printed samples, an outcome likely due to the increased surface roughness of the SLA samples compared to the UV chamber-cured samples, leading to potential applications for polymers typically considered too hydrophobic for tissue applications while also being a consideration if a polymer already presents ideal wettability. The SLA 3D printing process was also found to significantly increase the stiffness of PEGDMA samples compared to the UV chamber samples, opening an avenue through which the stiffness of a polymer can be increased for the purpose of cellular adhesion and differentiation. Taken together, SLA’s ability to impact both the wettability and stiffness of PEGDMA while maintaining consistent rates of polymerisation provide a promising approach to use in adapting PEGMDA properties for tissue engineering applications, which could be extended to other UV-polymerised polymers.

## Figures and Tables

**Table 1:** Thermal properties of PEGDMA hydrogels prepared via UV chamber and SLA polymerisation determined by DMA.

Polymer	Mean Tg (°C)
PEGDMA (UV chamber) chamber)	−23.8 ± 1.4
PEGDMA (SLA)	−24.9 ± 1.5

**Table 2:** Thermal properties of PEGDMA hydrogels prepared via UV chamber and SLA polymerisation.

Polymer	Mean Tg (°C)
PEGDMA (UV chamber) chamber)	−42.5 ± 0.7
PEGDMA (SLA)	−41.7 ± 0.3
